# A Lightweight Distributed Framework for Computational Offloading in Mobile Cloud Computing

**DOI:** 10.1371/journal.pone.0102270

**Published:** 2014-08-15

**Authors:** Muhammad Shiraz, Abdullah Gani, Raja Wasim Ahmad, Syed Adeel Ali Shah, Ahmad Karim, Zulkanain Abdul Rahman

**Affiliations:** 1 Center for Mobile Cloud Computing (C4MCC), Faculty of Computer Science and Information Technology, University of Malaya, Kuala Lumpur, Malaysia; 2 Department of History, Faculty of Arts and Social Sciences, University of Malaya, Kuala Lumpur, Malaysia; Xiamen University, China

## Abstract

The latest developments in mobile computing technology have enabled intensive applications on the modern Smartphones. However, such applications are still constrained by limitations in processing potentials, storage capacity and battery lifetime of the Smart Mobile Devices (SMDs). Therefore, Mobile Cloud Computing (MCC) leverages the application processing services of computational clouds for mitigating resources limitations in SMDs. Currently, a number of computational offloading frameworks are proposed for MCC wherein the intensive components of the application are outsourced to computational clouds. Nevertheless, such frameworks focus on runtime partitioning of the application for computational offloading, which is time consuming and resources intensive. The resource constraint nature of SMDs require lightweight procedures for leveraging computational clouds. Therefore, this paper presents a lightweight framework which focuses on minimizing additional resources utilization in computational offloading for MCC. The framework employs features of centralized monitoring, high availability and on demand access services of computational clouds for computational offloading. As a result, the turnaround time and execution cost of the application are reduced. The framework is evaluated by testing prototype application in the real MCC environment. The lightweight nature of the proposed framework is validated by employing computational offloading for the proposed framework and the latest existing frameworks. Analysis shows that by employing the proposed framework for computational offloading, the size of data transmission is reduced by 91%, energy consumption cost is minimized by 81% and turnaround time of the application is decreased by 83.5% as compared to the existing offloading frameworks. Hence, the proposed framework minimizes additional resources utilization and therefore offers lightweight solution for computational offloading in MCC.

## Introduction

Recent developments in mobile computing technology have changed user preferences for computing. Smart Mobile Devices (SMDs) have replaced a number of portable computing and communication devices as all-in-one device [1], [2]. Human dependency on the smartphones is increasing in different fields of life including e-business, e-education, entertainment, gaming, management information systems, and healthcare [3]. The consumer and enterprise market for cloud based mobile applications is expected to raise $9.5 billion by 2014 [4], which predicts the growth of applications for Mobile Cloud Computing (MCC). SMDs are predicated to employ computational intensive applications identical to the station based computers [5]; however, mobile applications on the latest generation of smartphones and tablets are still constrained by battery power, CPU potentials and memory capacity of the SMDs [6]. Therefore, MCC is employed to leverage the services of computational clouds for mitigating resources limitations in SMDs [7], [8].

Computational clouds facilitate to increase the computing capabilities of resources constrained client devices by offering on demand access to the widespread services and resources of cloud datacenters [9]. Computational clouds offer different service models for the provisioning of computing services [10]. For example, Elastic Cloud Compute (EC2) is employed for application processing services and Simple Storage Service (S3) of Amazon Web Services (AWS) is utilized for off-device storage [9]. MCC employs the services of computational clouds for enabling computational intensive and ubiquitous mobile applications on SMDs. For instance, the application processing services of computational clouds are utilized for augmenting application processing potentials of SMDs. Recently, a number of computational offloading frameworks are proposed for enabling intensive mobile applications on SMDs [5]. For instance, Apple iCloud [11] and Amazon Silk [12] browser are two latest mobile applications which leverage the services of computational cloud for application processing.

Traditional computational offloading frameworks employ application partitioning and component migration for computational offloading to the computational clouds. Elastic mobile applications are partitioned at different granularity levels and the intensive partitions of the applications are migrated at runtime for computational offloading. Therefore, current frameworks involve the overhead of application partitioning and additional cost of transferring the application binary code and corresponding data file(s) of the running instances of mobile application to the remote server node. Existing computational offloading frameworks lack of considering the intensity of runtime application partitioning and component migration. Therefore, resources intensive platform is established at runtime for the distributed processing of intensive mobile application. Such frameworks result in larger data transmission cost, high energy consumption and longer turnaround time of the mobile applications in accessing the application processing services of computational clouds [5], [13]–[15]. The resources constrained nature of SMDs requires deploying lightweight procedures for leveraging the application processing services of computational clouds. Lightweight computational offloading techniques require minimal resources utilization on SMDs in accessing the application processing services of cloud server nodes [6]. Therefore, mobile users are enabled to utilize distributed services with lower computational load on mobile devices, shorter turnaround time of the application and relatively long lasting battery lifetime.

This paper presents a lightweight Distributed Computational Offloading Framework (DCOF) for computational offloading in MCC. DCOF employs distributed approach for the configuration of intensive mobile application between mobile device and cloud server node. It eradicates the overhead of application partitioning and component migration at runtime, as a result the amount of data transmission, energy consumption cost and turnaround time of the application is reduced in cloud based processing of mobile application. The framework is evaluated by testing prototype application in real MCC environment. The lightweight nature of the proposed framework is validated by comparing results of employing DCOF and latest computational framework [16]–[18] for computational offloading in MCC. Analysis of the results shows that by employing DCOF for computational offloading the size of data transmission is minimized 91%, energy consumption cost is reduced 81% and turnaround time of the application is decreased 83.5%. Hence, the proposed framework minimizes resources utilization in leveraging the application processing services of computational clouds and offers lightweight procedure for computational offloading in mobile cloud computing.

The paper is classified into the following sections. Section 2 discusses related work in computational offloading for MCC. Section 3 presents the architecture of proposed framework and explains the operating procedure of DCOF. Section 4 describes methodology used for the evaluation of proposed framework. Section 5 presents results and discusses experimental findings. Finally, section 6 draws concluding remarks and future directions.

## Related Work

In the recent years, a number of cloud server based application offloading frameworks are introduced for outsourcing computational intensive components of the mobile applications to cloud datacenters [5]. Elastic applications are partitioned at runtime for the establishment of distributed processing platform. The offloading frameworks for MCC employ static or dynamic application partitioning mechanism. The static application partitioning mechanism [19] involves single time application partitioning for the distribution of workload between SMD and cloud server node, wherein the intensive components of the application are partitioned and transferred to the remote server node. For example, the primary functionality offloading [20] mechanism involves partitioning and offloading of the intensive components at runtime. Static application partitioning is simple mechanism for the distribution of computational load; however, it lacks of coping with the dynamic processing load on SMDs. Dynamic application partitioning [21]–[23] involves runtime profiling mechanism for determining the intensive components of the application which need to be offloaded to the clouds server node. Dynamic application partitioning is a robust technique for coping with the dynamic processing loads on SMD. Current dynamic partitioning approaches analyze the resources consumption of SMDs, computational requirements of the mobile application and search for runtime solving of the problem of resource limitations on SMD [24].

A number of frameworks employ Virtual Machine (VM) migration based computational offloading, wherein the running instance of mobile application is encapsulated in the virtual machine image [13]. It includes creation of VM instance, encapsulation of the running mobile application in the VM instance and transmission of the VM image on the wireless medium to the remote server node. On the cloud server node, a fresh VM instance is created and the delegated VM instance is cloned onto the newly created VM instance. Mobile application resumes its running state and application is executed on remote server node. However, VM migration based computational offloading requires additional computing resources for the deployment and management of VM and migration of VM instance to remote server node [13]. As a result, the execution cost and turnaround time of the application is increased. Furthermore, the migration of running application along with its data and active states is susceptible to security breaches and attacks.

Computational offloading is composed of three phases including initialization, computational offloading and remote application execution. (a) In the initialization phase, the availability of services on the cloud server node are discovered, context information reports are collected from various sensor modules. Furthermore, application characteristics such as security level and QoS demands are also gathered. The information collected in this phase is used for the offloading mechanisms. (b) The computational offloading process involves decision of application partitioning and offloading of an application, user authentication and authorization, VM instance creation on mobile and cloud server, migration of VM clone, QoS parameter negotiation and resources reservation. (c) Once the delegated application is configured, the running state of the application is resumed on the remote virtual device instance and application is executed on remote server node. Recently, a number of mobile cloud applications employ cloud computing to alleviate resources constraints of SMDs. For instance, Apples iCloud [11] provides applications such as music, photos, apps, calendars, documents automatically on demand basis. Apples iCloud employs the PaaS (Microsoft Azure) and IaaS (EC2) of Amazon for hosting the application store. Similarly, Silk application [12] is released by Amazon, which is a cloud-accelerated web browser. Silk is a split browser which resides on both Kindle Fire and EC2. For each web page request, Silk dynamically determines distribution of computational load between the local SMD and remote Amazon EC2. Silk considers the objective functions of network conditions, page complexity and the location of any cached content.

Existing frameworks [16], [22], [23], [25] employ application partitioning and component migration for computational offloading to the cloud server nodes. Mobile applications are partitioned at different granularity levels and the intensive partitions of the applications are migrated at runtime for computational offloading [24]. The mechanism of runtime application partitioning and component migration results in longer turnaround time of the application and larger size of data transmission. The timing cost of runtime computational offloading includes preferences saving time (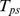), binary code offloading time of the application (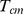), time taken in uploading the data states of the mobile application to remote server node (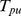), application download time to remote virtual device instance on the cloud server node (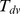), application reconfiguration and resuming time on the remote server node (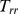), remote application execution time (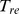) and time taken in returning the resultant data file to local mobile device (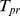). Therefore, the turnaround time of a single component of the mobile application which is offloaded at runtime is given by equation (1).
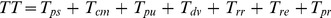
(1)


The Size of Data transmission (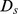) in runtime computational offloading involves the size of application binary file migrated at runtime (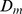), the size of preferences file uploaded to cloud server node (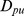) and the size of resultant preferences file downloaded to the local (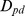). The total size of data transmission of a single component of the mobile application which is offloaded at runtime is given by equation (2).
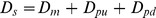
(2)


Therefore, current frameworks [16], [22], [25]–[30] involve the overhead of application partitioning and additional cost of transferring the application binary code and corresponding data file(s) of the running instances of mobile application to the remote server node. As a result, a resources intensive and time consuming distributed platform is established for the distributed processing of intensive mobile applications.

## Proposed Distributed Computational Offloading Framework (DCOF)

We propose as a lightweight alternative for the processing of intensive mobile applications in MCC. DCOF enables intensive mobile applications on the SMDs and reduces the additional overhead of computational offloading to the cloud server nodes. DCOF aims at leveraging the application processing services of cloud datacenters with minimal resources utilization on SMD. DCOF employs the SaaS model of computational clouds for accessing the services of cloud server nodes on demand basis. It focuses on dynamic computational task offloading to the cloud server node instead of dynamic intensive partition migration. The configuration of resources intensive components of the mobile application on the cloud server nodes results in eradication of the overhead of transmitting the application binary files and data files to the cloud server node at runtime. Computational load of the intensive mobile application is distributed by eliminating the overhead of migrating application binary file and active states of the application at runtime.

However, relying on the preconfigured services of the cloud server nodes lead to the problem of dependency on the centralized services and reduced offline usability. Similarly, it leads to the employment of thin client applications such as traditional web and email applications, wherein the processing logic of the application is hosted on the remote server nodes and client applications provide user interface. In order to address such issues, the proposed framework employs replication of the intensive components of the mobile application on mobile device and cloud server node. DCOF employs two distinct operating modes (offline mode and online mode) for the execution of mobile application. The offline mode of the application execution indicates an ideal situation wherein, sufficient computing resources are available on the local mobile device for the execution of mobile application. Therefore, in the offline mode all the components of mobile application are enabled to be scheduled for execution on the local mobile device. The profiler mechanism dynamically evaluates availability of resources (RAM, CPU and battery power) and future demands of the execution of mobile applications on SMD. Mobile application switches to online mode in the critical condition wherein the application processing services of computational clouds are used for the execution of intensive components of the mobile application. The required input data are transmitted to the cloud server node and upon the successful execution of the task on the cloud server node, resultant data are returned back to mobile device.

**Figure 1:**
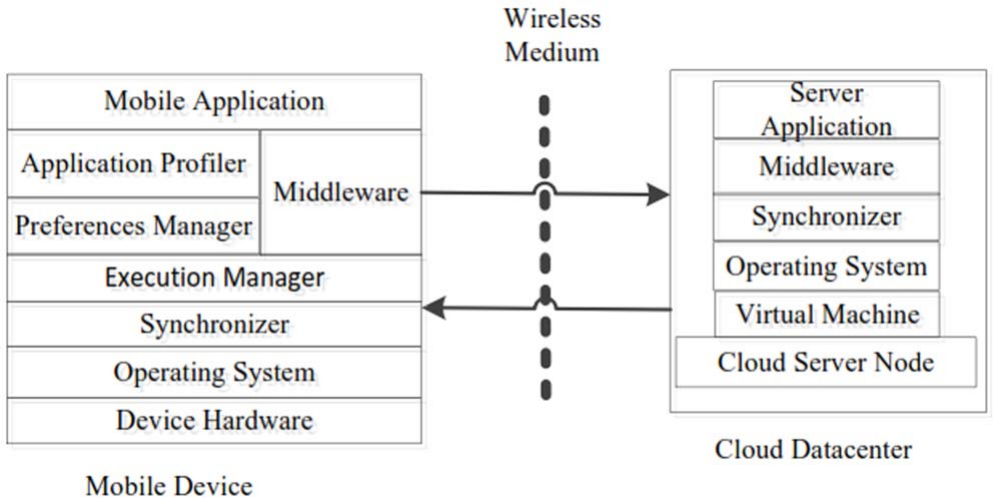
Architecture of the Proposed Distributed Computational Offloading Framework.

DCOF implements method level granularity for computational task offloading. Traditional computational offloading frameworks employ additional library which is coupled with the compiler support for tagging the intensive components of the mobile application (as remote) at compile time. Such frameworks [22], [23], [25], [31]–[33] involve the overhead of code generator module which is executed against the modified code, takes the source le as input and generates necessary remote-able method wrappers and utility functions. The remotely tagged methods are used as potential candidate methods for computational offloading. However, DCOF does not require the annotation of individual methods of the mobile application as local and remote. Therefore, DCOF reduces the developmental efforts for the distribution of execution load between mobile device and cloud server node. Furthermore, DCOF eliminates the additional overhead of distributed application deployment for leveraging the application processing services of cloud datacenters in MCC. Fig 1 shows architecture of the proposed framework.

The computational intensive components of the mobile application which do not require users interaction are configured on the cloud server node, which is provided access on demand basis in the online mode of application execution. DCOF based mobile application is based on the conventional application framework for mobile devices. However, mobile application is enabled to switch dynamically between online and offline mode. The dual operation modes of the mobile application enables to dynamically switch between online and offline mode. Applications are capable to operate with full functionalities on local mobile device in the situations of remote server access problems. Application is enabled to operate as a standalone application in the offline mode and to access distributed cloud services in the online mode. DCOF is composed of the following components.

### Application Profiler

Application profiling mechanism is implemented for automating the mechanism of application partitioning and computational offloading [24]. Prolers are important part of computational offloading frameworks. Computational offloading frameworks employ different types of profilers. For instance, application profiling dynamically evaluates availability of resources (CPU, RAM) on mobile device and computational requirements of mobile application [25]. Similarly, network profiling mechanism determines accessibility of network and quality of signal strengths while accessing the wireless access medium in MCC. Energy profiler examines utilization of battery power during the processing of mobile application. Memory profiler assesses the memory allocated to applications running on the mobile device and availability of memory for future allocations [22], [34]. The accurate and light weight nature of the profiling mechanism results in correct decisions for computational offloading [25], [35]. Profiling mechanism is significant for the reason that it determines the feasibility of application partitioning and component offloading. Profiler decides the destination location for the execution of mobile application. Based on the objective function considered by the computational offloading framework, profiler decides either to execute the component locally or remotely. DCOF employs application profiling for dynamically switching between online and offline mode of execution. Application profiler dynamically evaluates resources utilization on SMD and it works in coordination with the execution manager for switching the application between online and offline modes.

### Execution Manager

Execution manager monitors the execution modes of the mobile application. In the critical conditions, application is switched to the online mode wherein the running instance of the component of the application is terminated after saving the running state of the application. Execution manager is responsible for the configuration of the mobile application on SMD in the dual operating mode. In the online mode execution manager enables mobile application on the local device to access the services on the cloud server node for remote application processing, whereas in the offline mode all the components of the application are executed on local mobile device. Preferences Manager: The execution manager component is assisted by the preferences manager component for saving data states of the running instance of the application (in the online mode) and resuming the active state of the application on the local mobile device (in the offline mode). Preferences manger provides access to the preferences file of the application. Active data state of the application is written to the preferences while switching to the online mode. Similarly, data is read from preferences file while switching back to the offline mode of the execution. Synchronizer component of DCOF accesses the preferences for the exchange of data files between SMD and remote server node.

### Synchronizer

The synchronizer component of DCOF monitors synchronization of data transmission between local mobile device and cloud server node. It is responsible for the synchronization of the distributed components of the application. Synchronizer provides different types of services in the online mode of the application execution. Synchronizer coordinates for the synchronization between the application running on local mobile device and the application running on the cloud server node.

### Middleware

Mobile applications require distributed services access features and the configuration of middleware services for enabling access to the distributed services of cloud server nodes. DCOF provides a transparent distributed application execution environment in the online mode. In the online mode the services of distributed middleware are employed for computational task offloading. The execution manager saves the execution states of the identified intensive component by using preferences manager component of DCOF framework. The running instance of the executing component is terminated and the allocated resources are released to reduce the execution load on local mobile device. DCOF employs middleware services for accessing the services of cloud server node. Application running on mobile device activates the services of cloud server node by employing Inter Process Communication (IPC) mechanism such as RPC or RMI [6]. Mobile devices implement different middleware services [36] based upon the operating system platform. Middleware hides the complications of the communication between the local mobile application and cloud server application. DCOF provides a transparent distributed application execution environment in the online mode and therefore, mobile users are provided the notion as all the components of the mobile application are executed locally on SMD. For instance, we employ kSOAP2 API [37] for accessing the application processing services on the cloud server node. kSOAP2 is a lightweight SOAP client library for the Android platform [38]. Similarly, we employ Web Service Definition Language (WSDL) [39] middleware on the cloud server node for enabling access to the services of cloud server node.

**Figure 2:**
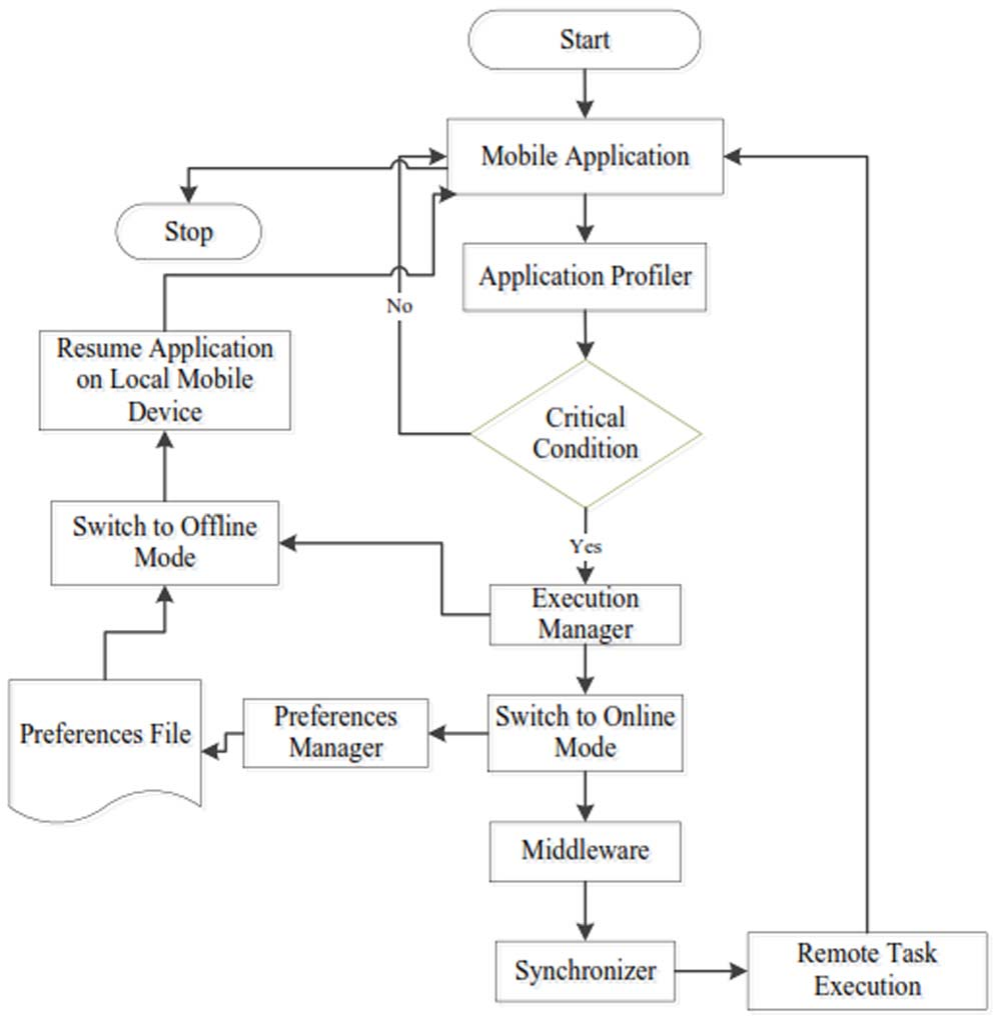
Illustration of the Interaction of the Components of EECOF Framework in POP and SOP.

Fig 2 shows the flowchart for the interaction of the components of DCOF framework in the execution of mobile application. Mobile application is employed on the mobile device and is capable to access cloud server node for computational task offloading. The application profiler evaluates resources utilization on mobile device continuously. In the critical conditions (for instance low battery), application execution manager switches the application to the online mode. Execution manager considers the execution history of the running instances of the components of mobile application for making the decision of computational offloading. The running states of the component of the application which is executing for a longer period of time and which utilizes abundant processing potential of the mobile device are saved by using the preferences manager. Mobile application accesses the services of cloud server node and the required input parameters are transferred for remote application execution. It is important to highlight that only the resources intensive tasks of the application are offloaded to the cloud server node, whereas the rest of the application continues execution on the mobile device. The synchronizer component of DCOF is responsible for the synchronization between mobile application running on the local mobile device and components of the application running on the cloud server node. Once the computational task is performed successfully on the cloud server node, final results are returned to the application running on mobile device. The services of the cloud server node always remain in the active mode. In the meanwhile, whenever remote services become inaccessible for the reason of interruption in network connectivity, execution manager is capable to switch back to the offline mode and to resume the running state of the application on local mobile device. However, the decision of resuming to the offline mode is based up on the feasibility of application execution on local mobile device. Execution manager makes the decision of resuming running state of the interrupted component on the basis of the input from the application profiler.

To reduce the dependency on the remote server node, DCOF involves the replication of computational intensive components of the application on mobile device and cloud server node. Application replication involves the complexities of consistency and synchronization in the distributed processing of the application between mobile device and remote cloud server node. However, the replication of intensive components of the mobile application assists in achieving the goals of minimal resources utilization in computational offloading, rich user experiences and offline usability. Resources utilization is reduced by eliminating the overhead of runtime application partitioning and partition migration at runtime. Similarly, rich user experience and offline usability is ensured by dynamically switching mobile application between online and offline mode. Therefore, application on the mobile device can resume the interrupted running state of the intensive component of the application in the situations of unavailability of remote services on the cloud server node.

DCOF employs traditional client/server architecture in the online mode of the application. However, the architecture and operation procedure of DCOF are different from the traditional client/server applications. The traditional client/server applications are thin client applications, wherein client applications are dependent on the server component of the application. The client applications provide user agents for interaction with the local computer and the processing logic is implemented on the server machines. Examples of such applications include web application, email application, social network applications such as Facebook, and video conferencing applications such as Skype application. Therefore in the traditional client/server model, client component of the application becomes inactive in the situations of inaccessibility of the server application. DCOF addresses such issues by enabling the operation of mobile application in two distinct modes. The application on the mobile device remains operational even though the services of computational clouds are inaccessible. Similarly, the components of the application can be executed on the local mobile device in the online mode.

## Evaluation

This section discusses the methodology used for the evaluation of proposed framework and explains experimental findings.

### Methodology

#### Experimental Setup

The proposed framework is evaluated by testing the prototype application for Android devices in the real mobile cloud computing environment. The server machine is configured for the provisioning of services to the mobile device in the online mode. SaaS model of computational clouds is employed for the provision of services to mobile devices. Mobile device accesses the wireless network via Wi-Fi wireless network connection of radio type 802.11 g, with the available physical layer data rates of 54 Mbps. Java based Android software development toolkit (Android SDK) is deployed for the development of the prototype application. Power Tutor tool [40] is used for the measurement of battery power consumption in distributed application processing.

#### Prototype Application

The Service Oriented Architecture (SOA) of Android application framework is employed for the development of prototype application. The prototype application is composed of three computational intensive components. (a) Sorting service component implements the logic of bubble sort for sorting liner list of integer type values. The sorting operation is tested with 30 different computational intensities (11000–40000). (b) The matrix multiplication service of the application implements the logic of computing the product of 2-D array of integer type values. Matrix multiplication logic of the application is tested with 30 different computational intensities by varying the length of the 2-D array between 160*160 and 450*450. c) The power compute service of the application implements the logic of computing b ∧ e, whereas b is the base and e is the exponent. The power compute logic of the application is tested for 30 different computational intensities by varying the exponent between 1000000 and 200000000. Empirical data are collected by sampling all computational intensities of the application in 30 different experiments and the value of sample mean is shown with 99% confidence for the sample space of 30 values in each experiment.

Data are collected by running the prototype application in three different scenarios. In the first scenario, the components of the mobile application are executed on the local mobile device to analyze resources utilization and turnaround time of the application on mobile device. In the second scenario, the intensive components of the mobile application are offloaded at runtime by implementing the latest techniques [16], [18] which involve entire component migration for computational offloading in MCC. In this scenario, we analyze size of data transmission and turnaround time of the mobile application. In the third scenario, DCOF is employed for evaluating resources utilization on mobile device and turnaround time of the application in cloud based application processing. The evaluation parameters include RAM allocation on mobile device (MB), CPU utilization on mobile device (MIPS), the size of data transmission (KB), and Turnaround Time (TT) of the application (ms). RAM allocation shows the amount of memory allocated to a particular component of the application on mobile device. CPU utilization indicates the percent CPU utilization during the execution of the component of mobile application on mobile device. TT parameter represents the total time taken in the execution of the component of mobile execution. The size of data transmission parameter represents the amount of data transmitted over the wireless network medium for offloading the components of mobile applications. The amount of data transmission affects the cost (energy consumption and turnaround time) of computational offloading for MCC.

### Results and Discussion

This section discusses experimentation findings of evaluating DCOF by employing the prototype application. It presents analysis of turnaround time, amount of data transmission, and energy consumption cost of the application from the perspective of local and remote application execution. Remote execution of the application is evaluated by employing the latest component offloading frameworks [16], [18] and DCOF based computational offloading for MCC.

As shown in the equation (1) the turnaround time (TT) of each component of the application in traditional computational offloading includes preferences saving time (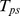), binary code offloading time of the application (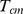), time taken in uploading the data states of the mobile application to remote server node (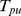), application download time to remote virtual device instance on the cloud server node (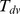), application reconfiguration and resuming time on the remote server node (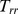), remote application execution time (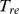) and time taken in returning the resultant data file to local mobile device (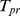). However, DCOF employs computational task migration rather than application partitioning and intensive components migration at runtime. Therefore, the TT of each component of the application in DCOF based computational offloading involves time taken in task offloading (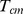), remote application processing time (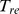) and preferences download time (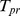).
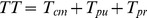
(3)


**Figure 3:**
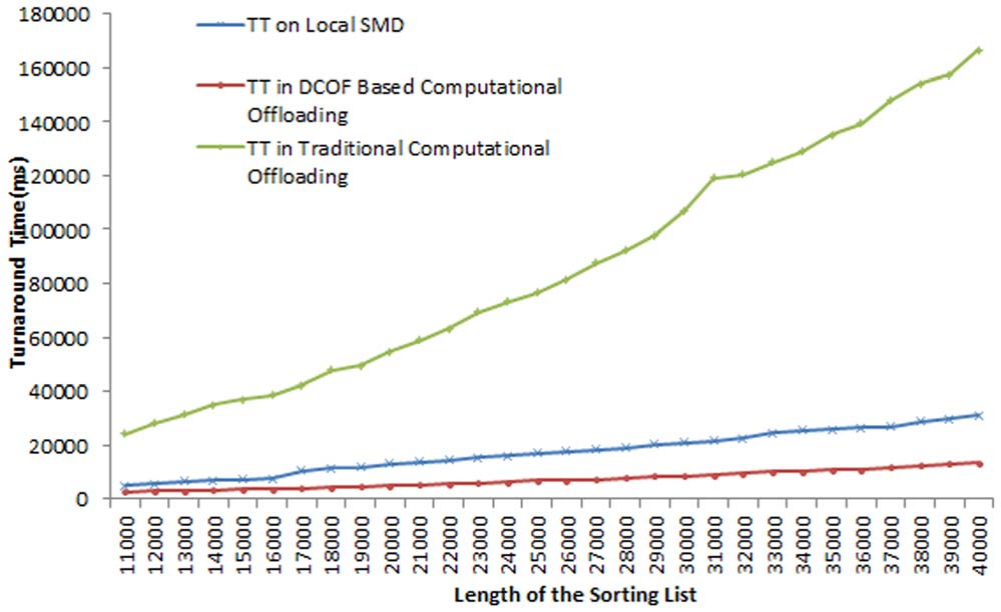
Comparison of the Turnaround Time of the Sorting Service Execution in Local and Remote Execution.

It shows that DCOF eliminates the additional delay incurred during component migration (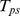, 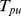) and reconfiguration (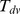, 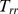) on the remote server node. Therefore, the TT of the intensive operation is reduced in each instance of computational offloading of the prototype application. Fig 3 shows the comparison the TT of sorting service execution in different scenarios. It is found that for sorting service execution the TT and resources utilization on SMD varies with the varying intensities of the sorting operation. For instance, the TT is found 4876 ms for list size 11000, 16950 ms for list size 25000, and 31207 ms for list size 40000. We found that TT for sort service execution on SMD increases by 84.3% for sorting list of 40000 values as compared to sorting list of 11000 values. The comparison of TT for sorting operation in local execution and traditional offloading techniques shows that TT of the sorting service increases considerably in runtime component offloading. It is observed in offloading sorting service component the TT of the sorting operation increases by: 80% for sorting list of 11000 values, 75% for sorting list 17000 values, 80% for sorting list of 30000 values and 81% for sorting list of 40000 values.

The comparison of sorting service execution on local mobile device and the DCOF based computational offloading shows decrease in TT of the sorting operation in the online mode of DCOF. We examined that by accessing the services of cloud server on in the online mode of DCOF, the TT of sorting services reduces by: 48% for sorting list of 11000 values, 60% for sorting list of 25000 values and 57% for sorting list of 40000 values. The overall reduction in TT value for sorting service is found by 57.8(+/−) 2% with 99% confidence in the sample space of 30 values. The comparison of TT for the sorting operation in the DCOF based computational offloading and traditional offloading shows the lightweight nature of DCOF framework for computational offloading. The decrease in DCOF based offloading of sorting service as compared to traditional runtime component offloading is found 89% for sorting list of 11000 values, 91% for sorting list of 20000 values, 92% for sorting list of 31000 values and 92% for sorting list of 40000 values.

**Figure 4:**
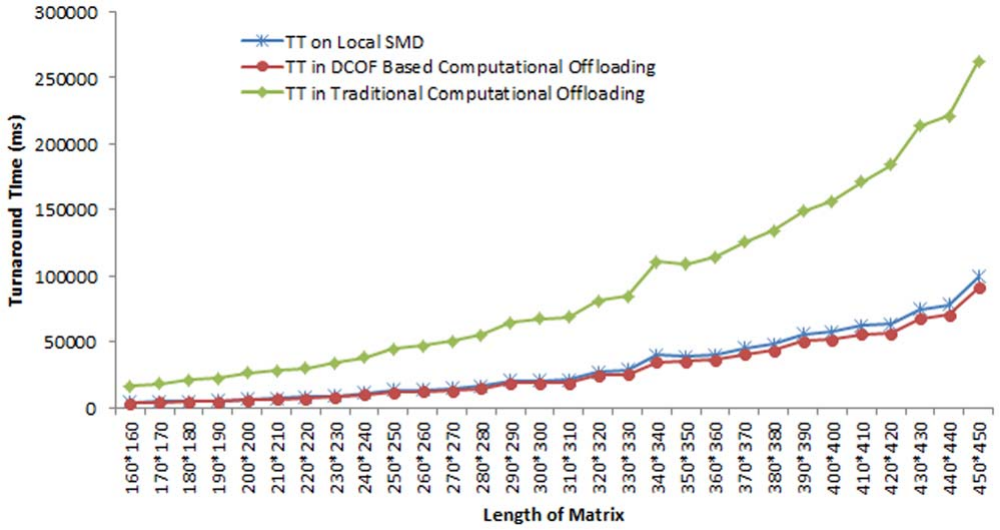
Comparison of the Turnaround Time of the Matrix Multiplication Service Execution in Local and Remote Execution.

Fig 4 shows the comparison of the TT of matrix multiplication service execution in different scenarios. It is observed that for matrix multiplication operation the execution time is 3653 ms for matrices length 160*160, 21185 ms for matrices length 310*310 and 99286 ms for matrices length 450*450. It shows that the execution time increases 96.3% for multiplying matrices of length 450*450 as compared to matrices of length 160*160. TT of the matrix multiplication increases considerably in runtime component offloading. It is observed in offloading matrix multiplication service the TT of the matrix multiplication service in remote processing compared to local execution on mobile device increases by: 78% for multiplying matrices of length 160*160, 70% for multiplying matrices of length 250*250, 66% for multiplying matrices of length 300*300 and 65% for multiplying matrices of length 450*450.

DCOF based computational offloading however, reduces the TT of the matrix multiplication operation as compared to both local and traditional computational offloading based execution. It is observed that DCOF based computational offloading reduces the TT of matrix multiplication operation by: 10% for matrices of length 160*160, 9% for multiplying matrices of length 350*350 and 8% for multiplying matrices of length 450*450. The overall reduction in TT for matrix multiplication service in DCOF based offloading of matrix multiplication operation is found 10.3(+/−) 0.5% with 99% confidence in the sample space of 30 values. The decrease in TT of matrix multiplication operation in DCOF based offloading as compared to traditional runtime component offloading is examined 74% for multiplying matrices of length 160*160, 72% for multiplying matrices of length 230*230, 64% for multiplying matrices of length 350*350 and 63% for multiplying matrices of length 450*450.

**Figure 5:**
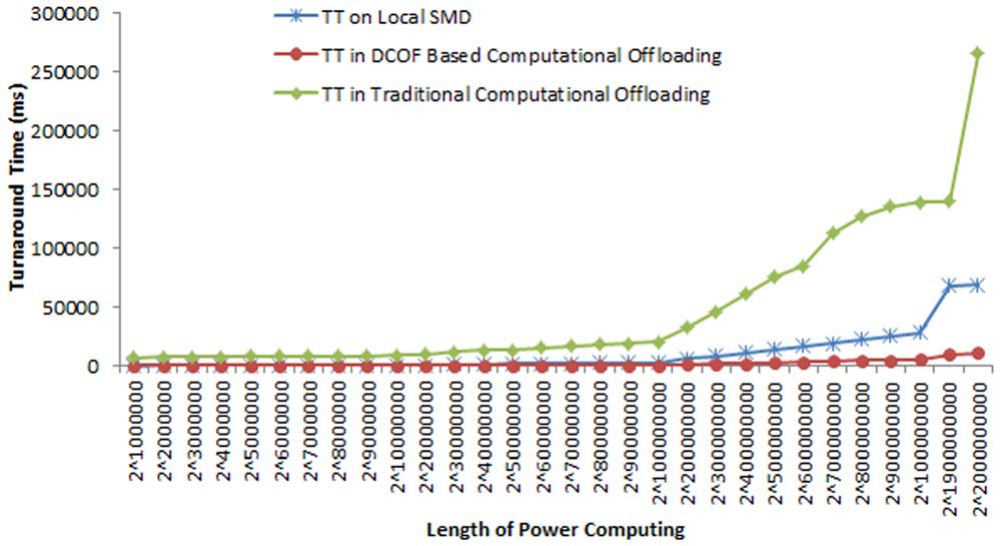
Comparison of the Turnaround Time of Power Compute Operation in in Local and Remote Execution.

Fig 5 shows the comparison of turnaround time of the power compute service execution for local and remote execution. It shows that the turnaround time of power compute operation increases with the increase in the intensity of power compute operation. For example, the TT on local mobile device is found 51 ms for computing 2 ∧1000000, 1767 ms for computing 2 ∧60000000 and 69044 ms for computing 2 ∧2000000000. It shows the TT for power compute service execution increases by 99.9 for computing 2 ∧2000000000 as compared to computing 2 ∧1000000. The comparison of TT for power compute operation in local execution and traditional offloading technique shows that TT of the power computing increases considerably in runtime component migration. We found that in runtime component offloading the TT of power computing increases by: 99.3% for computing 2 ∧1000000, 96.2% for computing 2 ∧20000000, 81.4% for computing 2 ∧400000000 and 74% for computing 2 ∧2000000000.

The comparison of TT of power compute operation in local and remote execution shows that DCOF based computational offloading is insignificant for lower intensities of the application. For instance, the TT of power compute operation for computing 2 ∧1000000 is 33% higher in DCOF based computational offloading as compared to the local execution of power compute operation. It is for the reason of additional delays incurred in the process of computational offloading. However for higher intensive operations, DCOF based computational offloading reduces the TT of the power compute operation as compared to both local execution and traditional computational offloading. It is observed that DCOF based computational offloading reduces the TT of power compute operation 6.3% for computing 2 ∧2000000, 47.7% for computing 2 ∧20000000 and 84.2% for computing 2 ∧2000000000. The overall reduction in TT in DCOF based offloading as compare to local execution of power compute operation is found 65(+/−) 10.8%. Similarly, the decrease in TT of power compute operation in DCOF based offloading as compared to traditional runtime component offloading is examined by: 99% for computing 2 ∧2000000, 98% for computing 2∧20000000 and 95.9% for computing 2 ∧2000000000. The overall reduction in TT of power compute operation in DCOF based offloading as compare to traditional runtime component offloading is found 97(+/−) 0.6%.

As shown in equation (2) the amount of data transmission in runtime computational offloading involves the size of application binary file migrated at runtime, the size of preferences file uploaded to cloud server node and the size of resultant preferences file downloaded to the local mobile device. However, the size of data transmission in DCOF based computational offloading of each component of the application involves the amount of data transmitted to remote server node as input parameter and amount of data transmitted as the final result returned from remote server node to the local mobile device. DCOF eliminates the additional data transmitted in migrating application binary code and preferences files. Therefore, the size of data transmission is reduced in each instance of computational offloading of the prototype application.

**Figure 6:**
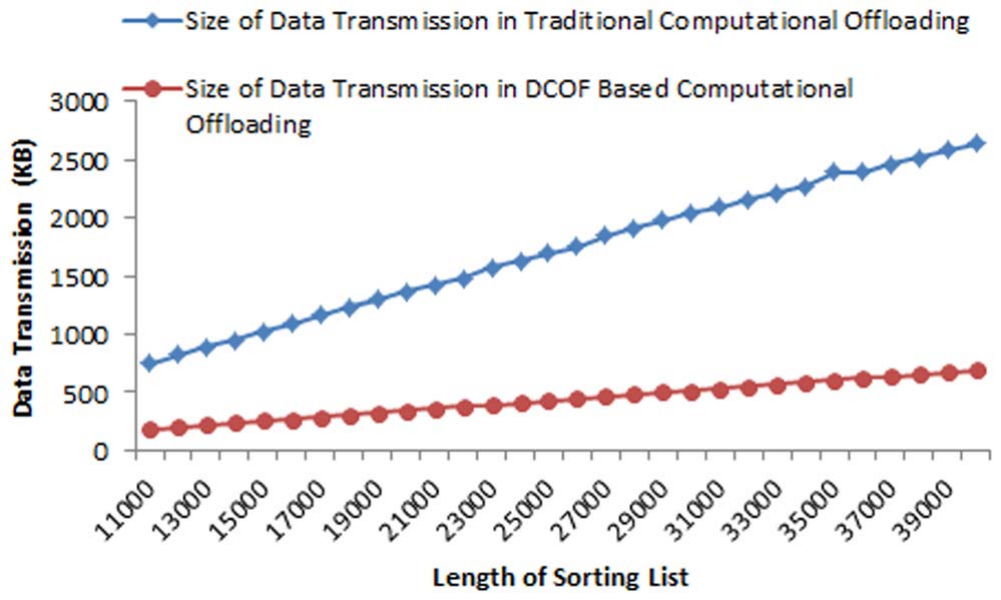
Comparison of the Size of Data Transmission in Traditional Offloading and DCOF Based Offloading for Sorting Operation.

Fig 6 shows the size of data transmission in offloading sort service component of the application at runtime. It is examined that in all instances of offloading sorting service, the size of binary application file (.apk) remains 44.4 KB for sort service; whereas, the size of preferences file uploaded to the cloud server node and the size of the resultant preferences file downloaded to the local mobile device varies for different intensities of the sorting operation. The size of data transmission for offloading sort service component with the list of 11000 values is found 752.4 KB, whereas the size of data transmission in accessing sorting service of DCOF server application is examined 83 KB. Similarly, the size of data transmission is examined 2645.4 KB for list of 40000 values in traditional computational offloading, whereas the size of data transmission by employing DCOF is found 692 KB. It shows that by employing DCOF based computational offloading; the size of data transmission is reduced 76% for sorting list of 1100 values and 74% for sorting list of 40000 values. The average reduction of data transmission is found 74.7% by employing DCOF based computational offloading for the sorting list of 11000–40000 values.

**Figure 7:**
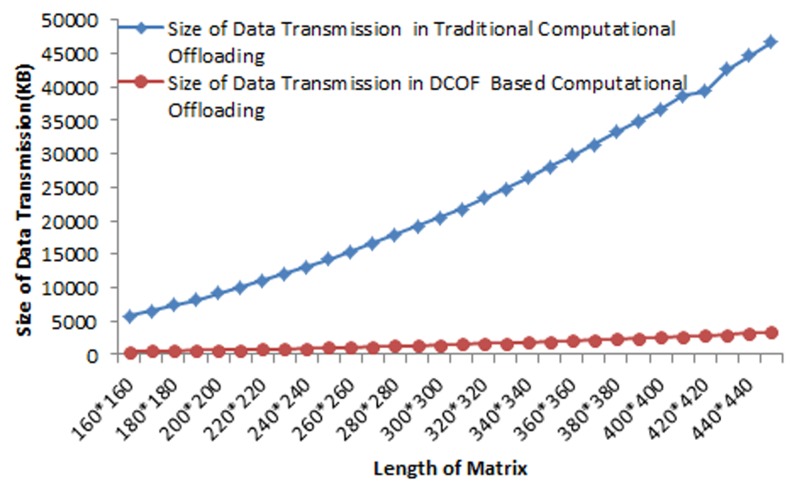
Comparison of the Size of Data Transmission in Traditional Offloading and DCOF Based Offloading for Matrix Multiplication Operation.

Fig 7 shows the comparison of data transmission in traditional runtime application offloading and proposed DCOF based computational offloading for matrix service component of the application. It is examined that in all instances of matrix multiplication service offloading the size of binary application file (.apk) remains 46 KB; whereas, the total size of data transmission in runtime offloading of matrix multiplication service is examined: 5739.4 KB for matrices length 160*160, 15426.5 KB for matrices length 260*260 and 46740 KB for matrices length 450*450. The average size of data transmission for offloading matrix multiplication service with the matrices length 160*160–450*450 is determined 11474.3 KB. However, the size of data transmission by employing DCOF for offloading matrix multiplication operation is found 463 KB for matrices of length 160*160, 1979 KB for matrices of length 350*350 and 3308 KB for matrices of length 450*450. It shows that the size data transmission is reduced by 91.9% for matrix size 160*160 and by 92.2% for matrix size of 450*450 values by employing DCOF for offloading matrix multiplication operation. The average reduction of data transmission over the wireless network medium is by 92% in DCOF based computational offloading for the matrices of size 160*160–450*450.

The size of data transmission for offloading power compute service by employing traditional computational offloading is evaluated in 30 different experiments. It is examined that in all instances of offloading power compute service the size of binary application file (.apk) is 42.7 KB; whereas, the size of preferences file uploaded to the cloud server node is 1 KB and the size of the resultant preferences file downloaded to the local is 1 KB. Therefore, the total size of data transmission is observed as 44.7 KB for offloading power compute service at runtime; whereas, the size of data transmission in employing DCOF for power compute service is found 2KB. It shows that the overhead of offloading the binary file of the power compute service is eliminated; therefore, the size of data transmission reduces 95.5% which results in the reduction of energy consumption cost and turnaround time of the application in cloud based application processing. The total size of data transmission in traditional computational offloading for the mobile application is observed as 24761 KB; whereas, the size of data transmission in DCOF based computational offloading is found 2074.6 KB. It shows that the amount of data transmission is reduced 95.6% in employing DCOF for offloading the intensive components of the application.

**Figure 8:**
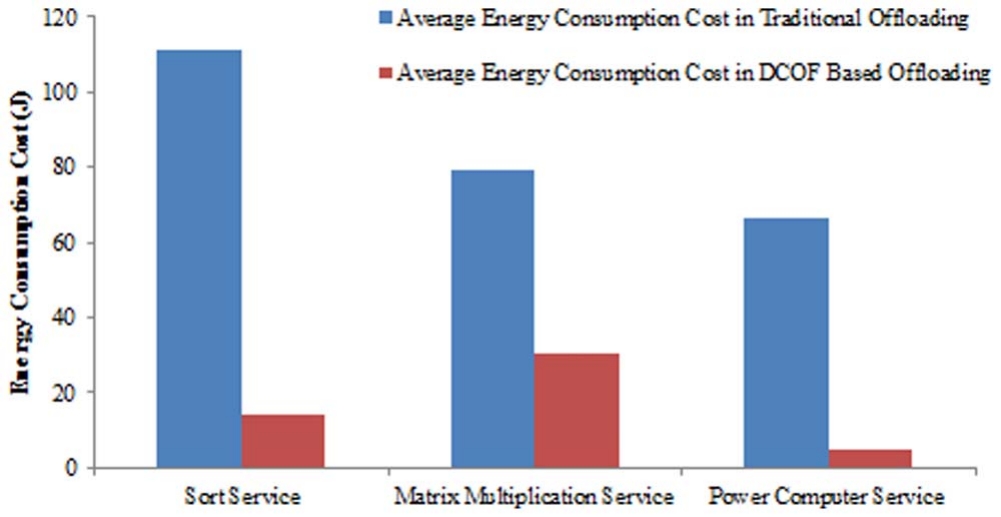
Comparison of Energy Consumption Cost in Traditional and DCOF based Computational Offloading.

The energy consumption cost is evaluated in 30 different experiments for all the three components of the application by employing traditional and DCOF base computational offloading. It is found that the energy consumption cost reduces considerably by implementing DCOF for offloading computational task to cloud server nodes. The decrease in the energy consumption cost is for the reason of reducing the overhead of additional resources utilization in the establishment of distributed application processing platform at runtime. Fig 8 compares the average decrease in energy consumption cost of offloading the components of the application in traditional and DCOF based computational offloading. It is examined that energy consumption cost reduces by: 85.1% for sorting 11000 values, 85.2% for sorting 20000 values, 87.8% for sorting 30000 values and 88.6% for sorting 40000 values.

The decrease in the energy consumption cost by employing DCOF for offloading sorting operation is found by 86.7% with 30 different intensities of the sorting operation. Similarly, it is examined that by employing DCOF based offloading, the energy consumption cost of matrix multiplication operation reduces by: 73% for matrices of length 160*160, 66.3% for matrices of length 300*300, 56.8% for matrices of length 400*400 and 50.4% for matrices of length 450*450. The average decrease in the energy consumption cost by employing DCOF for offloading matrix multiplication operation is found 64.3% with 30 different intensities of matrix multiplication operation. Similarly, the energy consumption cost of power compute operation reduces by 63% for computing 2 ∧1000000, 76.8% for computing 2 ∧30000000, 91.8% for computing 2 ∧200000000 and 96.8% in computing 2 ∧2000000000.

The employment of DCOF results in minimal resources utilization on SMD for computational offloading in MCC. It is observed that the additional cost of application binary code migration and active data state migration to the cloud server node is reduced by employing DCOF for cloud based processing of computationally intensive mobile applications. Therefore, turnaround time, size of data transmission and energy consumption cost is reduced in processing intensive mobile application on cloud server node. For instance, by employing DCOF the size of data transmission for sorting service is reduced by 74.8%, turnaround time of the sorting operation reduces by 91.3% and the energy consumption cost is reduced by 87.5% as compared to the traditional computational offloading technique [16], [18]. Similarly, the size of data transmission for matrix multiplication operation is reduced by 92.8%, turnaround time is reduced by 72% and energy consumption cost is reduced by 61.6% compared to the traditional computational offloading technique. In the same way, the size of data transmission for power compute operation is reduced by 95.5%, turnaround time is reduced by 97% and energy consumption cost is reduced by 93%.

## Conclusions

The mechanism of application partitioning at runtime and component migration increases data traffic, energy consumption cost and turnaround time of the application. Therefore, resources intensive and time consuming distributed application execution environment is established for computational offloading in MCC. DCOF is proposed to minimize the overhead of load distribution between mobile devices and cloud server node in leveraging the application processing services of computational clouds. DCOF employs lightweight operating procedures for computational offloading and leverages the SaaS model for the deployment of computationally intensive mobile applications in MCC. The incorporation of distributed services access technique for computational offloading facilitates in the optimal deployment procedure with minimal resources utilization for the establishment of distributed platform in MCC. The dual operating nature contributes to the versatility and robustness of the distributed framework for enabling intensive applications on resources constrained SMDs. Mobile applications are enabled to operate independently on the local mobile device in the offline mode; whereas, the services of computational clouds are employed on demand basis in the online mode for conserving computing resources of SMDs.

Analysis of the results signifies the lightweight nature of DCOF, which reduces the energy consumption cost, size of data transmission and turnaround time of the application in cloud based processing of the intensive component of mobile application. The additional cost of application binary code migration and active data state migration to the cloud server node is reduced by employing DCOF for computational offloading. It is found that by employing the DCOF for computational offloading, the size of data transmission is minimized by 91%, energy consumption cost is reduced by 81% and turnaround time of the application is decreased by 83.5% as compared to the contemporary offloading frameworks. Hence, the DCOF minimizes additional resources utilization and therefore offers lightweight solution for computational offloading in MCC. The future research includes extending the scope this research to address the issues of consistency of simultaneous application execution between local mobile device and remote cloud server node, and seamless application execution in leveraging application processing services of computational clouds in MCC.

## Supporting Information

Table S1 **Comparison of Application Processing Time (ms) of the Sort Service Component.**
(XLSX)

Table S2 **Comparison of Application Processing Time of Matrix Multiplication Service.**
(XLSX)

Table S3 **Comparison of Application Processing Time (ms) on SMD in Processing Application locallay and by implenting the POP of DEAP Model.**
(XLSX)

Table S4 **Summary of Data Transmission in Traditional and Proposed Framework Based Computational Offloading.**
(XLSX)

Table S5 **Summary of Data Transmission in Traditional and Proposed Framework Based Computational Offloading.**
(XLSX)

Table S6 **Comparison of ECC.**
(XLSX)
